# Strata behavior at fully-mechanized coal mining and solid backfilling face

**DOI:** 10.1186/s40064-016-3281-3

**Published:** 2016-09-20

**Authors:** Wei Yin, Zhiwei Chen, Kai Quan, Xiancheng Mei

**Affiliations:** School of Mining, China University of Mining and Technology, Xuzhou, China

**Keywords:** Fully-mechanized coal mining and solid backfill, Strata movement, Strata behavior, Backfill body

## Abstract

Taking Ping Dingshan Coal Mine Group 12 as an example, this paper explains the system layout, key equipment and backfilling technology in detail. It probes into the characteristic of rock strata movement behavior and surface deformation above the gob area through in-site measurement method. The results show that as the overburden strata are effectively supported by the backfill body in mined out areas, there were no evident phenomena as first weighting or periodic weighting during mining process. Besides, influencing scope of advanced support pressure and the strata behavior degree were much smaller than that of the traditional methods of caving mining. Since overburden strata had been well supported by backfill body, it shows the posture of sinking slowly, only resulting in bending zone and minor fracture zone.

## Background

The “three-under” (namely coal resources trapped under buildings, railways, and water bodies) accounts for a significant portion of coal reserves in China and, seriously restricts the recovery rate. According to an uncompleted statistics, ‘Three-unders’ coal storage amounted to 13.79 billion t only in state-owned coal mines, which are mainly concentrated in the eastern mining area. And the quantity of coal underlying buildings reaches 9.468 billion tons, which makes up 69 % of the total trapped quantity, and almost exists in every mine in China. Generally, the pressure coal takes 10–30 % of the recoverable reserves, and even all of the resources are ‘three-unders’ coal in some coal mines (Zhang et al. 2013). Most coal mines in eastern China have entered an exhausted stage when the resources are being mined out after decades of intensive mining (Miao et al. 2010a; Miao and Qian 2009). Domestic and foreign scholars have done a lot of research and practice on it. Varieties of backfill mining methods such as solid backfill, pneumatic backfill, cemented paste backfill and high-water material backfill have been put forward (Tapsiev et al. 2009; Belem and Benzaazoua 2008; Seryakov et al. 2008; Donovan and Karfakis 2004; Rankine and Sivakugan 2007). One of the most extraordinary among them applied in China is the fully-mechanized backfill mining (FMBM) (Ju et al. 2009; Zhang and Miao 2006).

In this technology, solid material is filled into the gob and then tamped as the working face advances. Acting as a permanent supporting body, the tamped solid material changes the characteristic of strata movement and behavior, surface deformation (Huang et al. 2010; Ju et al. 2009). It not only greatly improves the recovery rate of coal resources and facilitates solid waste disposal, but also protects the ecological environment of mining area and helps to achieve scientific and green mining (Qian et al. 2006) of coal resources (Miao et al. 2010b; Huang et al. 2011a). Although a lot of theoretical researches have been done by some scholars regarding the characteristic of strata movement and surface deformation, the law of strata behavior after backfill mining, it is based on a small portion of field data which is limited to support resistance of hydraulic support (Zhang et al. 2010a; Miao et al. 2010c), influencing scope of advanced support pressure (Zhang et al. 2010b; Miao et al. 2010d), the relationship between physical and mechanical characteristics of backfill material and roof subsidence (Zhang et al. 2010c; Huang et al. 2011b), etc., little efforts have been made to study mechanical characteristic of backfill materials in the gob, and its influence on strata behavior as well as the characteristic of strata movement and surface deformation after backfill mining. Taking Ping Dingshan Coal Mine Group 12 as an example, this paper details system layout, backfill mining technique, key equipment, characteristic of strata behavior and surface deformation in an attempt to promoting FMBM application (Huang et al. 2012).

## Mining conditions of backfill mining face

### General situation of backfill mining face

#### Geological conditions

**Fig. 1:**
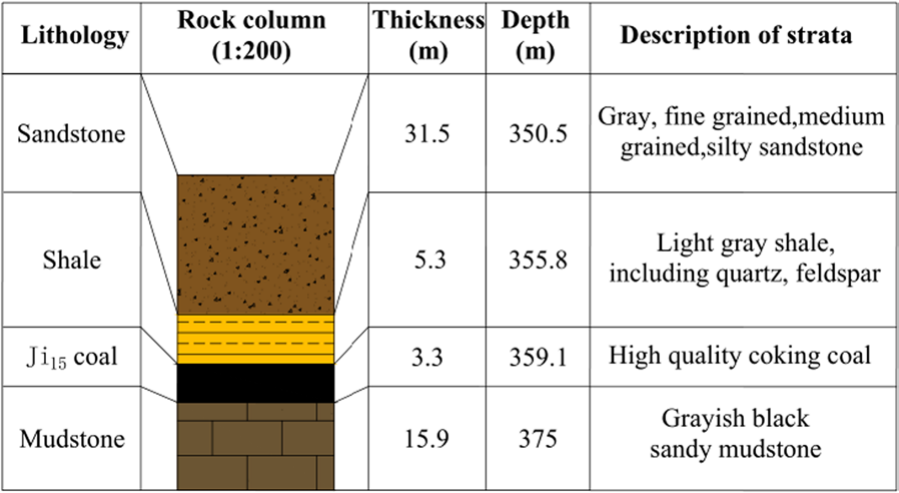
Roof and floor lithology of No 13080 backfill panel

No 13080 backfill panel, with an incline width of 100 m, an advancing length of 350 m and a recoverable reserve of 1.24 million tons, is the first backfill mining face in Ping Dingshan Coal Mine Group 12. It extracts the 15# coal seam with an average inclination of 8°, an average thickness of 3.3 m and a mass density of 1.4 t/m^3^. The spontaneous combustion of the coal seam lasts for 3–6 months. Figure 1 shows the basic conditions of the roof and floor. Rock physical and mechanical properties test results as shown in Table 1.

**Table 1:** Physical and mechanical properties of rocks

Rock	Elasticity modulus/GPa	Tensile strength/MPa	Cohesion/MPa	Poisson’s ratio	Density/kN/m^3^
Sandstone	28	6.0	13	0.26	23.8
Shale	25	3.5	7	0.23	24.2
Ji15 Coal	9	1.5	4	0.33	14.3
Mudstone	23	7.0	12	0.27	25.5

#### Surface situation of No 13080 backfill panel

**Fig. 2:**
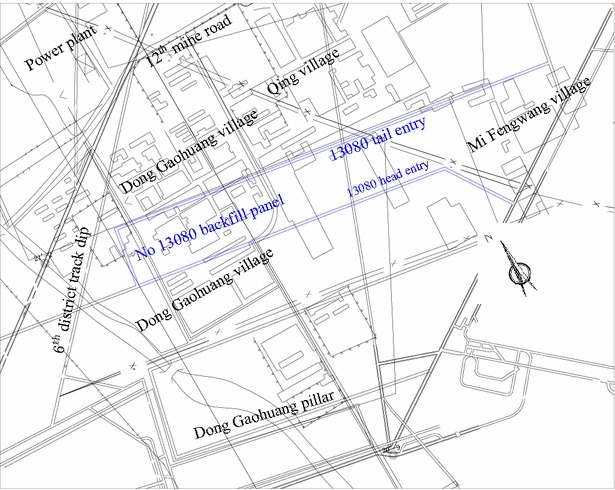
Position of No 13080 backfill panel and the corresponding surface condition

No 13080 backfill panel is located underneath the Dong Gaohuang and Mi Fengwang villages, the industry square of the Ping Dingshan Coal Mine Group 12 and Hezhuang Coal Mine. Figure 2 illustrates the position of No 13080 backfill panel and the corresponding surface conditions.

### System layout

**Fig. 3:**
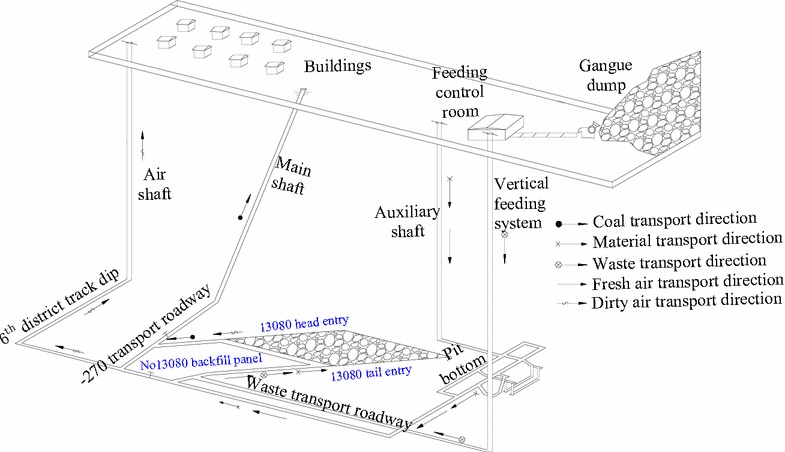
System layout of FMBM

The backfill material used at No 13080 backfill panel is gangue from a waste dump of in the east of industry square. The gangue is transported to the underground through vertical feeding system after screening and crushing. Figure 3 shows the system layout of FMBM.

### Key equipment for FMBM

Key equipment for FMBM includes backfill hydraulic support with six columns and a backfill conveyor with a bunch of unloading holes at the bottom, which provides the space for backfilling and mining and can also offer the backfill power. Hence, it ensures that the backfill and mining proceed in parallel.

#### Backfilling hydraulic support

**Fig. 4:**
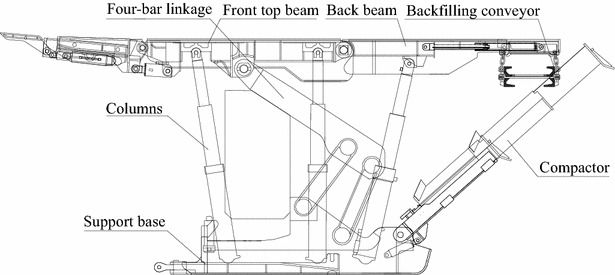
The structure principle of the support

The backfilling hydraulic support with six columns (type ZZC8800/20/38) is designed considering the geological condition and strata pressure, etc. It consists of a front top beam, six columns, a base, a four-bar linkage, a back beam and a compactor. Installed on the back of the support, the compactor presses solid material into the gob. Figure 4 illustrates the structure principle of the support, and the major technical parameters are shown in Table 2.

**Table 2:** Major technical parameters of support

Type	ZZC8800/20/38	Working resistance (kN)	8800
Height (mm)	2000–3800	Support strength (MPa)	0.73
Center distance (mm)	1500	Tamping force (kN)	1545
Initial supporting force (kN)	7824	Tamping angle (°)	0.50

#### Backfilling conveyor

According to the transport volume per hour of solid material needed for backfill and the type of support, backfilling conveyor (type SGZC764/250) has been designed. The basic parameters of backfill conveyor are shown in Table 3.

**Table 3:** The basic parameters of backfill conveyor

Type	SGZC764/250	Rated voltage (V)	1140
Transport ability (t/h)	500	Scraper chain type	Double center chain
Chain speed (m/s)	1.16	Chain spacing (mm)	500
Motor type	YBSD-200/100-4/8Y	Slot specification (mm)	1500 × 724 × 260
Rated power (kW)	200	Unloading hole size (mm)	345 × 240

### Backfilling and mining technology

The mining technology is the same as the traditional mining. Backfilling and mining work in parallel. Backfill proceeds from the tail to the head of the backfilling conveyor. After the material is accumulated to a certain height the next unloading hole is open and a machine rammer presses a tamping slab to tamp the material. This process would be repeated until the material has been sufficiently tamped and in general, it takes two or three cycles. The first round of backfilling would pause after the face has been fully filled. The backfilling conveyor then moves forward toward the head of the back beam and the compactor pushes all of the material that remains under the backfilling conveyor back and above till solid material reaches the roof. In this way, the material is being pushed toward the roof and compacted. Finally, close the unloading holes to back fill the space below the conveyor headpiece. After the first back filling cycle is completed, the conveyor would be pushed to the end of the back beam to start another cycle.

### The physical composition and mechanics property of backfill material

**Fig. 5:**
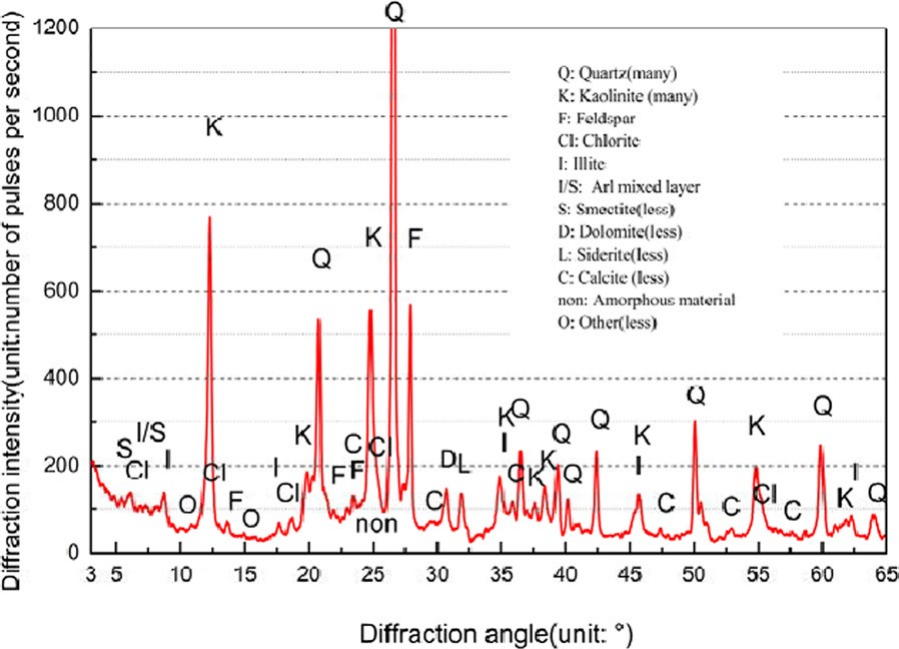
X-ray diffraction analysis of backfill material samples

The backfill material used at No 13080 backfill panel is gangue from a waste dump of in the east of industry square. The physical composition and mechanics property of backfill material could have a significant influence on the strata movement and ground subsidence. Hence, these parameters should be evaluated by laboratory test. The X-ray diffraction of D/Max-3B equipment is used in this test, The X-ray diffraction pattern is shown in Fig. 5, the physical composition and elements of backfill material samples are shown in Table 4.

**Table 4:** The physical composition and elements of backfill material samples/%

Samples	Na_2_O	MgO	Al_2_O_3_	SiO_2_	K_2_O	CaO	Fe_2_O_3_	P
Backfill material	0.65	1.8	17.6	59.9	2.4	2.0	5.4	0.06

S	F	Ba	Mn	Cu	Pb	Zn	Ti	Cl
0.44	≤0.045	0.10	0.054	<0.0002	<0.0002	0.009	0.50	0.011

According to Table 4, in the composition of backfill material samples, the content of SiO_2_ is the main component of backfilling materials frame, which makes the backfill materials in higher strength. Furthermore, owing to the contains materials of C, Al and CaO which make the backfill materials hydrolysis and weathering more easily.

**Fig. 6:**
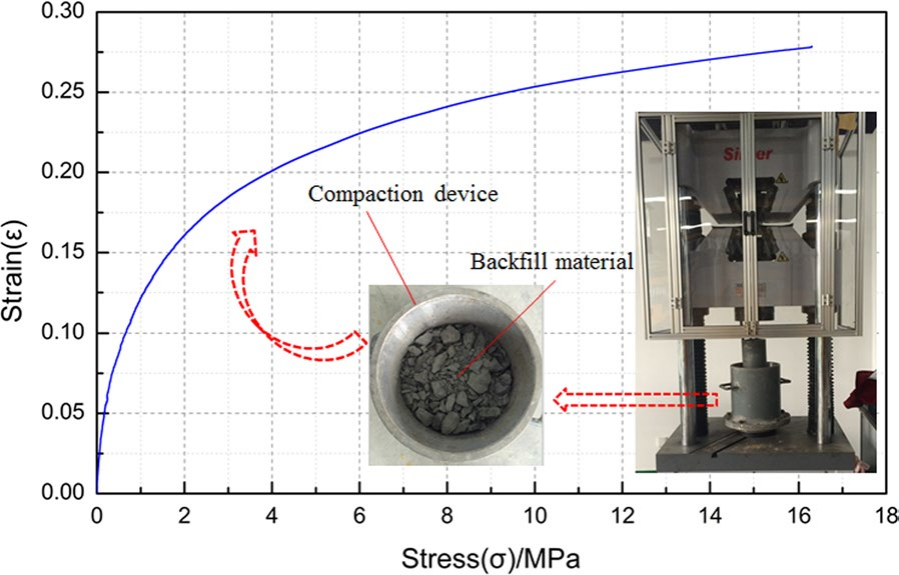
Strain and stress curves of backfill material

The campaction characteristics of backfill material were tested and the relationships between compaction force and deformation were also analysed. The tests are conducted by MTS815.02 electro-hydraulic servo rock mechanical testing system. The compaction device is an independent designed steel cylinder with the diameter of 230 mm, the wall thickness of 15 mm and the height of 180 mm. The stress–strain curve were illustrated in Fig. 6.

As shown in Fig. 6: the strain factor of back full material increases along with the increasing of loading stress; The relationship between the stress and stress is in logarithmic form. At the early stage of the compacting procedure when the loaded stress is smaller than 4 MPa, the increasing speed of material strain is high and 72 % of the overall compaction is finished during this stage; When the loading stress is over 4 MPa, the compacting speed decreases, the strain–stress curve becomes gentle, and the proportion of overall compaction occurs in this stage is <28 %.

## Strata behaviour at the panel

The strata behavior at No 13080 backfill panel had been measured, and major field data include: working resistance of the support, advanced abutment pressure in the coal, internal stress in the backfill material, dynamic subsidence of immediate roof in the gob and fracture development of overlying strata.

### The site measurement of working resistance

**Fig. 7:**
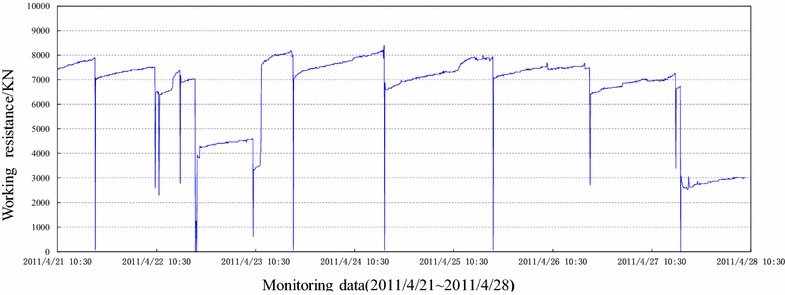
Working resistance distribution of backfill hydraulic support from 3# monitoring point

**Fig. 8:**
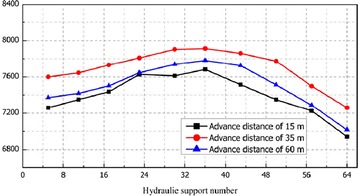
Average support pressure of the shields along inclined direction of the longwall face

Figure 7 illustrates the working resistance distribution of supports from 3# monitoring unit and Fig. 8 shows the average pressure distribution along inclined direction of working face.

The following conclusions can be drawn from Figs. 7 and 8:

1. In the whole process of mining, the supports’ working resistance stayed relatively stable and there had been no surge. The average working resistance varied from 7200 to 7800 kN which was much lower than the rated working resistance of 8800 kN, i.e. the safety valves were kept off. All these had not shown the evident phenomena of first weighting or periodic weighting. The reason was that backfill material carried most of the overburden pressure, hence, changed the structure of surrounding rock.
2. In the whole process of mining, the setting load of backfill hydraulic support varied from 6600 to 7000 kN which was slightly less than average working resistance of support. In this sense, backfill hydraulic support can effectively control the roof subsidence before backfilling.
3. The average working resistance of backfill hydraulic support reached 7400, 7700, and 7500 kN respectively when working panel advanced over 15, 35 and 60 m. The working resistance of support stayed stable in the whole process and the safety valves were kept off, which further proved that there existed no evident phenomena of first weighting or periodic weighting.

### The site measurement of advanced abutment pressure in the coal

**Fig. 9:**
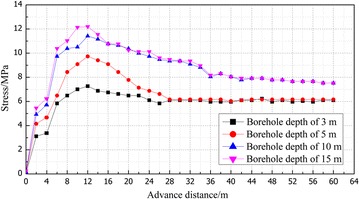
Advanced abutment pressure distribution curve for coal body of different depth

Advanced support pressure in the coal had been monitored by 24 borehole stress meters installed in the coal. The measured data from different borehole length are shown in Fig. 9.

Figure 9 illustrates that advanced support pressure in the coal can be divided into four stages:

1. The area within the range of 0–5 m of the distance from face is distressed zone because this area was being supported by backfill material, backfill hydraulic support and coal. The pressure value in this area was <2 MPa and the closer was to the coal wall, the smaller was the stress value.
2. The advanced abutment pressure values increase sharply in the coal within the range of 5–15 m of the distance from face. The peak value (12.2 MPa) appeared in the position which was 10–13 m away from the coal wall and the maximum stress concentration coefficient (the ratio of peak value and original stress, the field data show that the value of original stress is approximately 9.38 MPa) reached to 1.3. This area is classified as the stress increasing zone and the peak value zone.
3. The advanced support pressure values started to decrease in the coal within the range of 15–25 m from working face and this area is the stress decreasing zone.
4. When the distance was greater than 25 m, the stress in the coal approached the in situ stress gradually and this area is classified as the in situ stress area.
5. The scope of the stress increasing zone and the maximum stress concentration coefficient were smaller than those of traditional mining, which shows that FMBM would result in slighter strata behavior.

The above analysis shows that backfill materials filled in the gob had borne part of the overburden load. The peak value and the influencing scope of advanced support pressure are much smaller than those of the traditional ones.

### The internal stress of backfill body in the gob

**Fig. 10:**
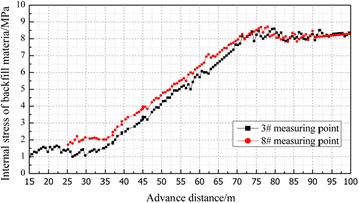
Stress curve of backfill material from 3# and 8# measuring points

The internal stress of backfill body in the gob is monitored by the stress monitors installed on the floor of gob. The vertical stress in the backfill material was measured and recorded and then transmitted to the surface via cables. The 3# and 8# measuring points were 20 and 40 m away from the open-off cut. The monitoring results are shown in Fig. 10.

1. The internal stress of backfill material changes over three stages as the working face advances. They are respectively the initial stress zone (0–35 m away from the working panel), the stress increasing zone (30–75 m away from the working panel) and the stable stress zone (75 m away from the working panel). And the change pattern of the internal stress of backfill material is consistent with the roof subsidence variation.
2. In the stage of initial stress zone, the internal stress varied from 1.1 to 2.2 MPa and kept basically stable, which illustrates that the roof subsidence in this zone was small.
3. In the stage of the stress increasing zone, the internal stress increased gradually but at a slow rate, which shows that the roof gradually bent and subsided and the backfill material was compacted step by step.
4. In the stable stress zone, the internal stress stabilized, peaking at 8.6 MPa which was close to the in situ stress. It implies that the bending and subsiding of the overlying strata tend to stop.

### The dynamic subsidence of immediate roof in the gob

**Fig. 11:**
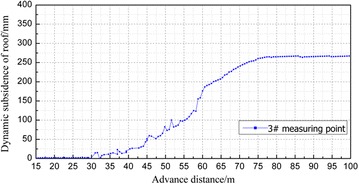
Dynamic subsidence curve of immediate roof from 3# measuring point

**Fig. 12:**
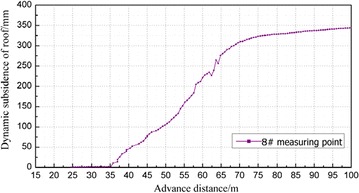
Dynamic subsidence curve of overlying strata from 8# measuring point

The roof subsidence in the gob is monitored by the roof sinking monitor installed on the floor of gob. The subsidence values in the backfill material were measured and recorded and then, transmitted through cable. The 3# and 8# measuring points were 18 and 38 m away from the open-off cut, respectively. The results are shown in Figs. 11 and 12.

1. The change pattern of dynamic subsidence of roof as the working face advances can be divided into three stages: the small deformation stage (0–35 m away from the working face), the deformation stage (30–75 m away from the working panel) and the stable deformation stage (75 m away from the working panel).
2. In the small deformation stage, the coal body behind the open-off cut and the coal body in front of the working panel bear most of the overlying strata load because the advancing distance is short. Therefore, the roof subsidence is small.
3. In the deformation stage, the coal body behind the open-off cut and the coal body in front of the working panel cannot offer enough force to support most of the overlying strata load as the advance distance increases. Therefore, the roof started to bend and subside, and the maximum values of 3# and 8# measuring points reached 267 and 340 mm respectively.
4. As the working panel continues to advance, the backfill material filled in the gob is compacted gradually and becomes the main body of supporting the overburden load. Hence, the roof subsidence stabilized. The final backfill ratios in 3# and 8# measuring points were 91.9 and 89.7 % respectively according to the maximum subsidence values in these two measuring points.

### The fracture development of roof in the gob

**Fig. 13:**
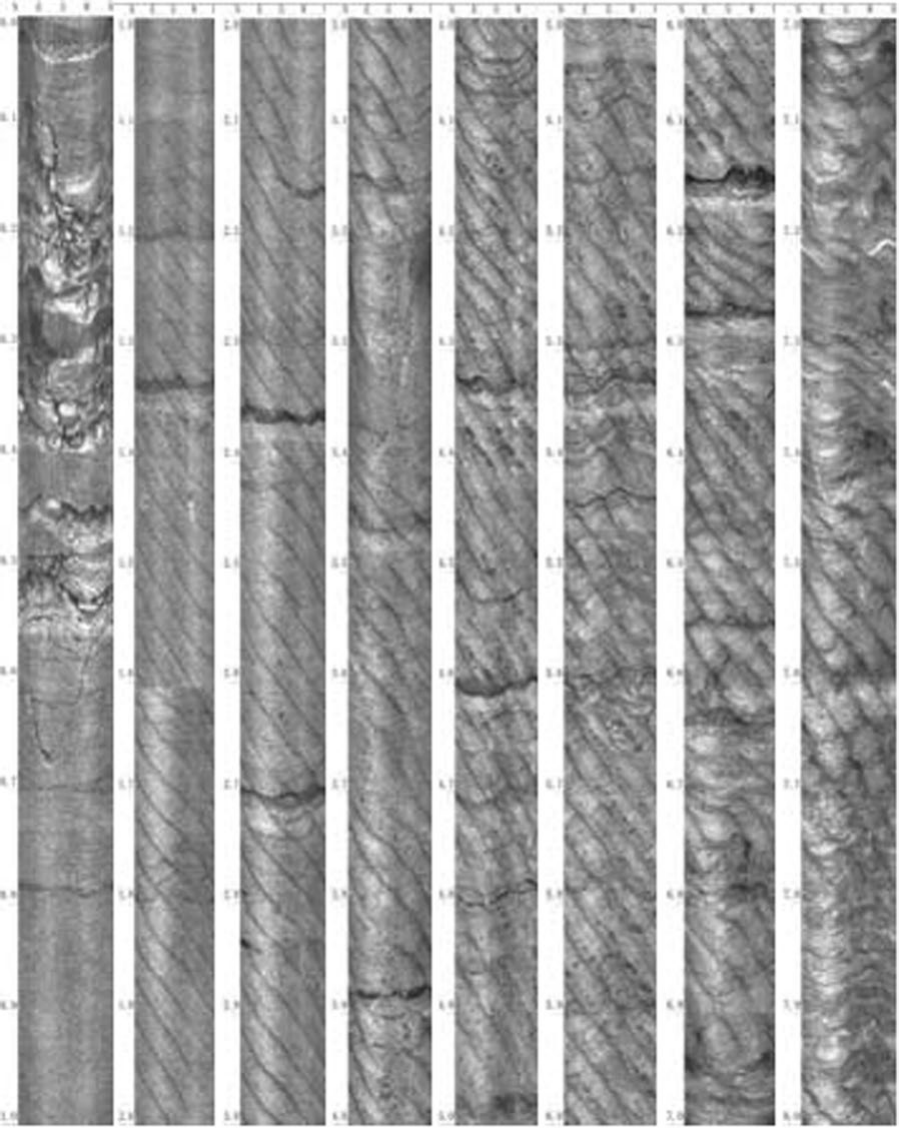
Shooting graphs from 1# drilling

The fracture development of roof is peeped through drilling boreholes towards the roof in the gob. The angle between borehole and vertical direction was 45° and the length of boreholes was 8 m. Figure 13 illustrates the peep pictures.

As the working panel advances, the strata subside as a whole and there is no occurrence of abscission. In the vertical direction, the layers close to the coal start to break and fractures begin to develop. As the working panel continually advances, fractures constantly expand upward, but the layers do not break. The conclusion can be drawn that the caving zone does not exist in the roof after backfill mining.

## Conclusions

1. The first weighting and periodic weighting phenomenon is not obvious since backfill material may carry the overburden pressure effectively.
2. The influencing scope of advanced support pressure and the strata behavior degree are much smaller than those of the traditional ones.
3. The overburden strata show the posture of sinking slowly, only resulting in bending zone and minor fracture zone since they are well and effectively supported by backfill body.
